# Supplementary material for: Peculiar features of the plastids of the colourless alga Euglena longa and photosynthetic euglenophytes unveiled by transcriptome analyses
Source: Sci Rep. 2018 Nov 19;8:17012. doi: 10.1038/s41598-018-35389-1 (PMC6242988; doi:10.1038/s41598-018-35389-1)
Supplement: Supplementary file 1 — Supplementary figures and Dataset S1 [file 41598_2018_35389_MOESM1_ESM.pdf]

Peculiar features of the plastids of the colourless alga *Euglena longa* and photosynthetic euglenophytes unveiled by transcriptome analyses

Kristína Záhonová, Zoltán Füssy, Erik Birčák, Anna M. G. Novák Vanclová, Vladimír Klimeš, Matej Vesteg, Juraj Krajčovič, Miroslav Oborník, Marek Eliáš

SUPPLEMENTARY FIGURES S1 – S8
SUPPLEMENTARY DATA S1

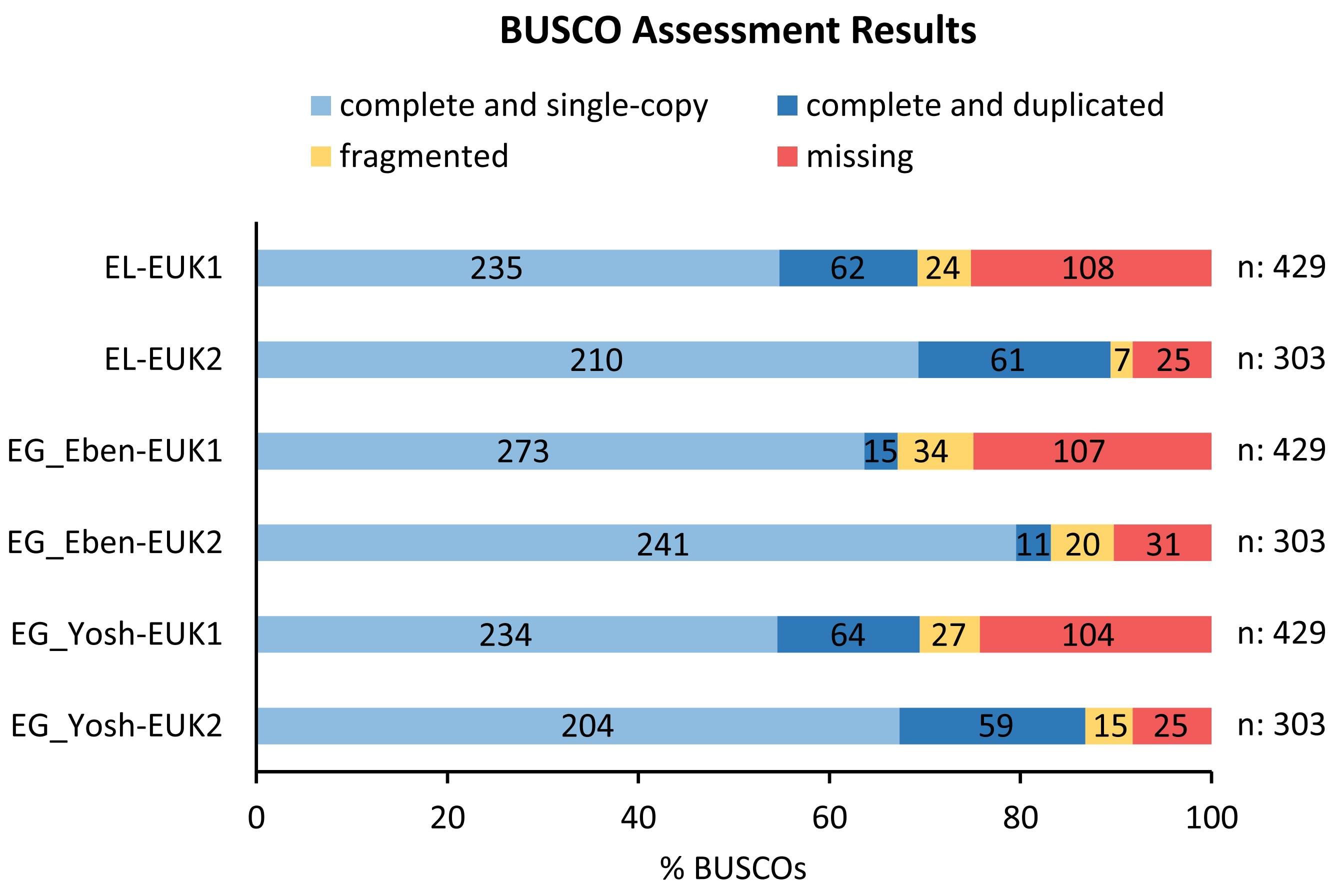

Figure S1: BUSCO analysis of the transcriptomic data from Euglena spp. Two sets of conserved eukaryotic orthologs (EUK1 and EUK2) were used to assess the completeness of the *E. longa* transcriptome, compared to publicly available data from *E. gracilis*. EG_Eben, *E. gracilis* from [7]; EG_Yosh, *E. gracilis* from [9]; EL, *E. longa* from this work; n, number of orthologs in the BUSCO dataset.

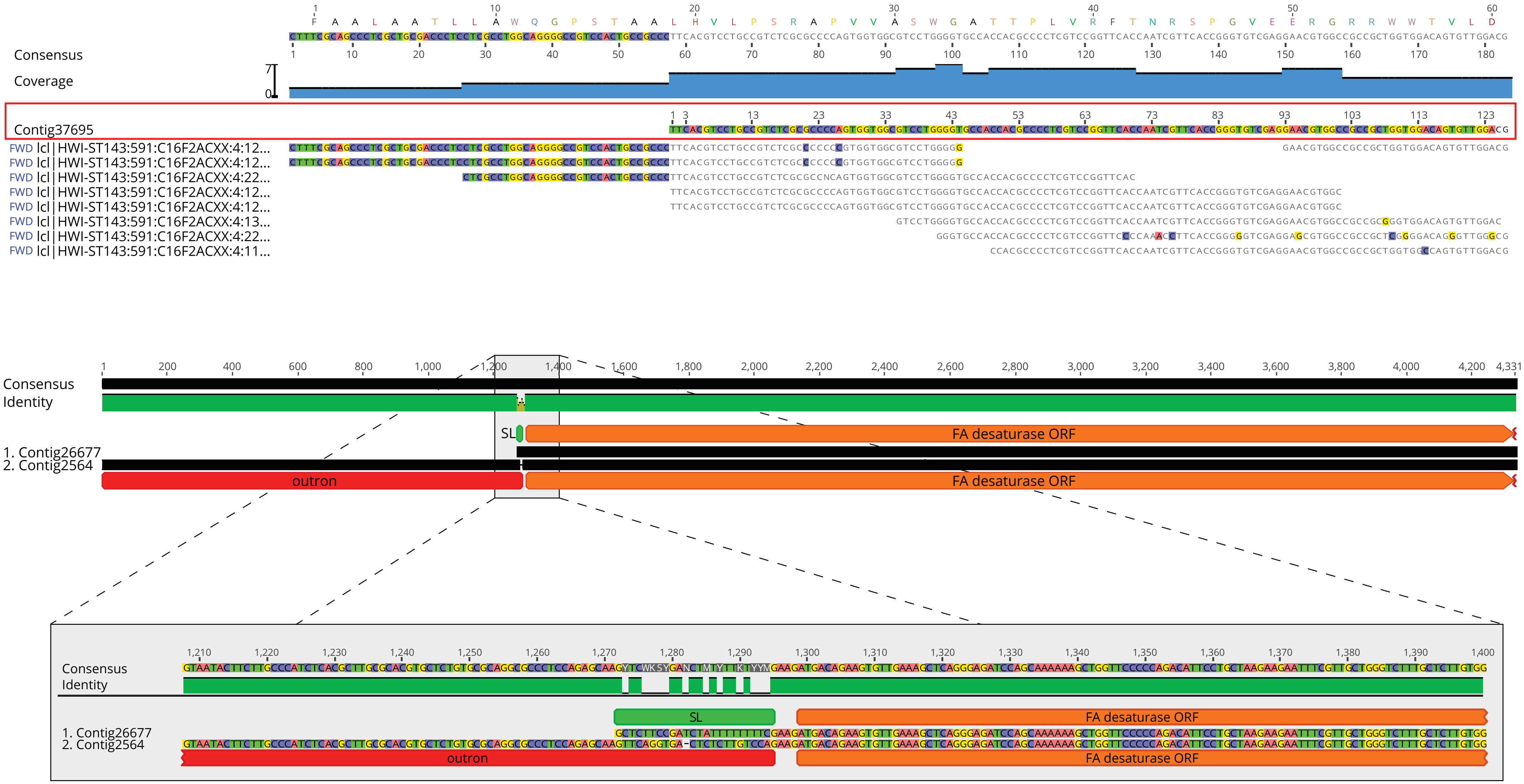

Figure S2: Examples of *E. longa* contigs lacking the 5'-end SL sequence due to truncation or incomplete splicing. A) Although iterative read mapping extended the assembled Contig37695 (arginyl-tRNA synthetase) towards its 5'-end, it did not yield the full open reading frame (indicated by protein sequence above the consensus sequence). B) Alignment of two contigs encoding the same protein (fatty acid desaturase; orange box) is presented. Contig26677 bears a SL sequence (green box) at its 5'-end, whereas Contig2564 represents the same transcript before trans-splicing of the outron (red box) and the SL-RNA.

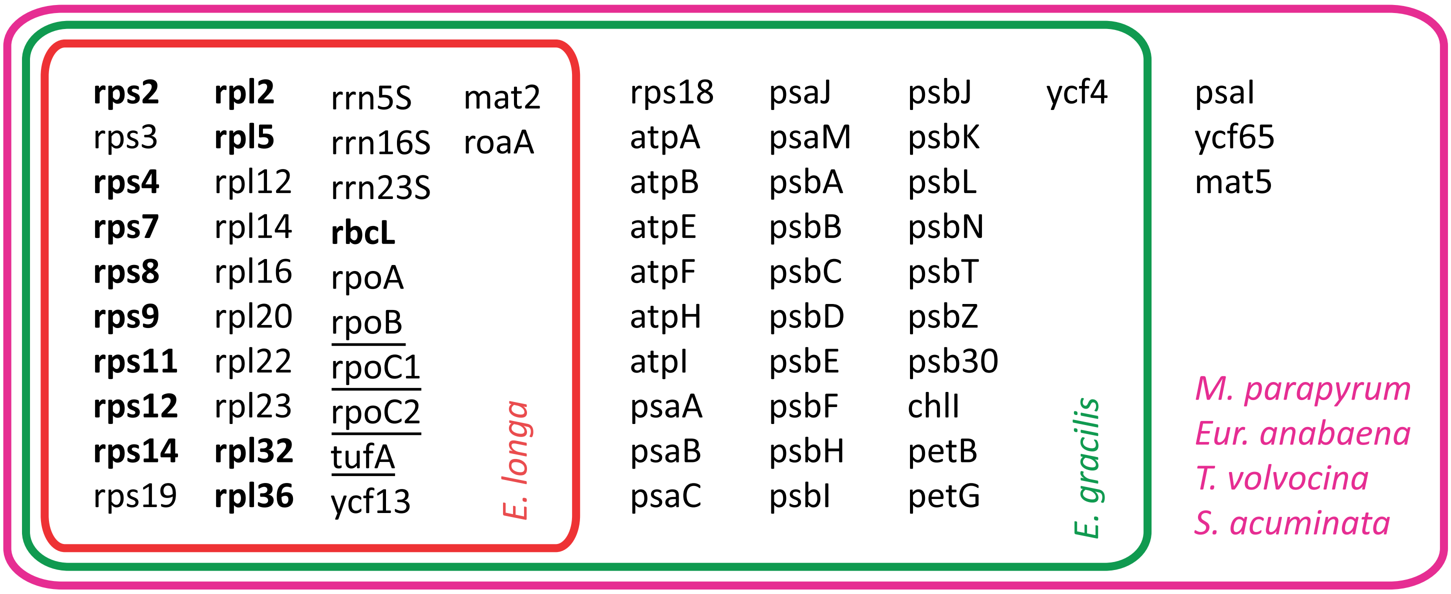

Figure S3: Genes in euglenophyte plastid genomes. For simplicity, only selected euglenophytes (*E. longa*, *E. gracilis*, and species with the maximum plastid-encoded genes found) are included. *E. longa* genes fully covered by the transcriptomic data obtained are in bold, genes with only a partial coverage are underlined.

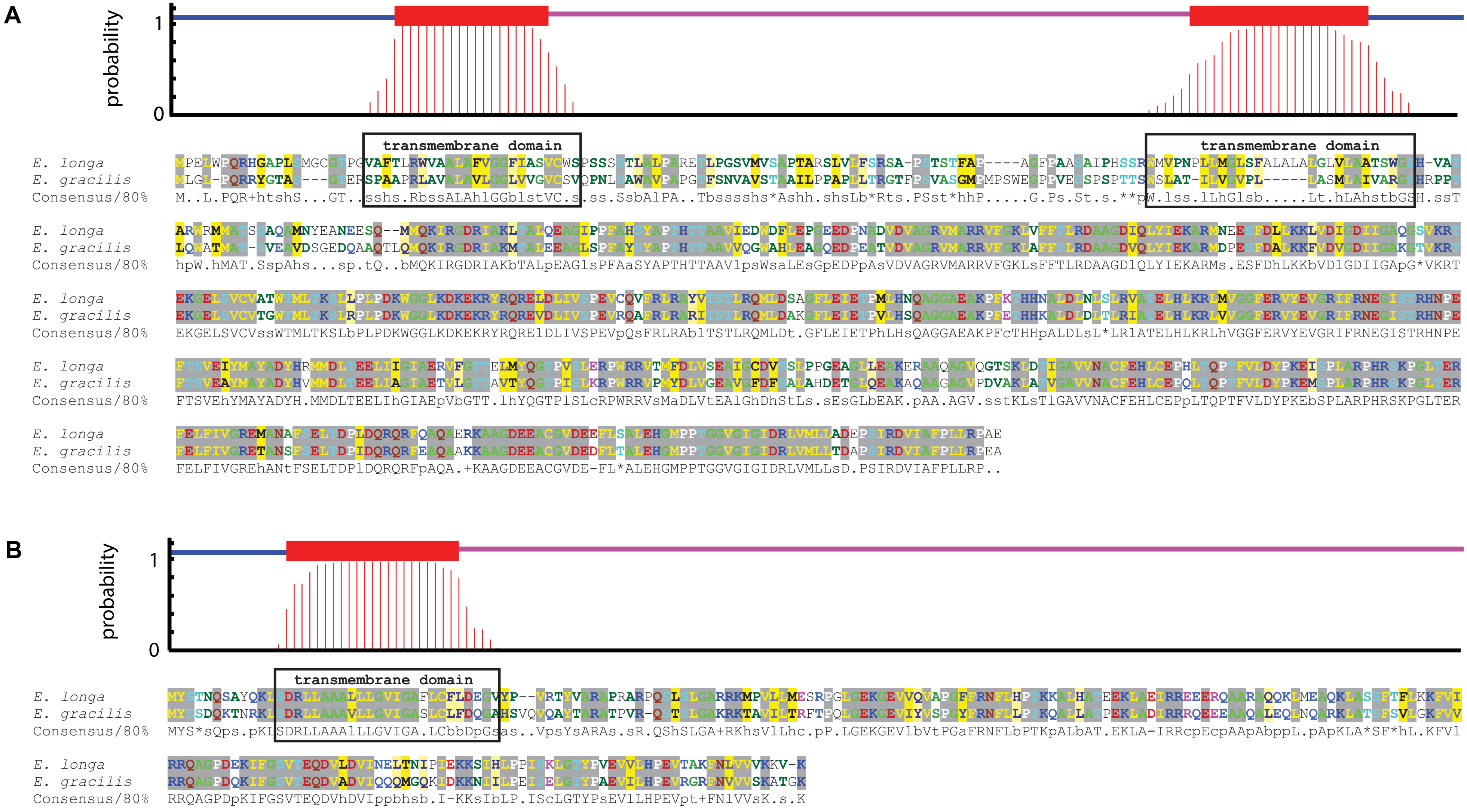

Figure S4: Two classes of plastid-targeting bipartite topogenic signals (BTSs) in *Euglena*. The figure shows two proteins exemplifying the two classes of plastid-protein presequences (or BTSs) that can be found in *Euglena* spp. The alignments were prepared using MAFFT v7 and processed for visualization using Chroma v1.0. Class I BTS (A; represented by lysyl-tRNA synthetase) contains two transmembrane domains (boxed), while class II (B; represented by ribosomal protein L9) has one. The first domain overlaps with the signal peptide, and is trailed by the transit peptide that is (Class I) or is not (Class II) followed by the second transmembrane domain presumably serving as a membrane anchor during the ER-to-plastid trafficking. Following the two-step cleavage of the presequence, the mature protein is released to the stroma (or possibly inserted into the inner envelope membrane via the so-called stop-transfer pathway). Note the high sequence similarity between the presequences of orthologous proteins from *E. gracilis* and *E. longa*.

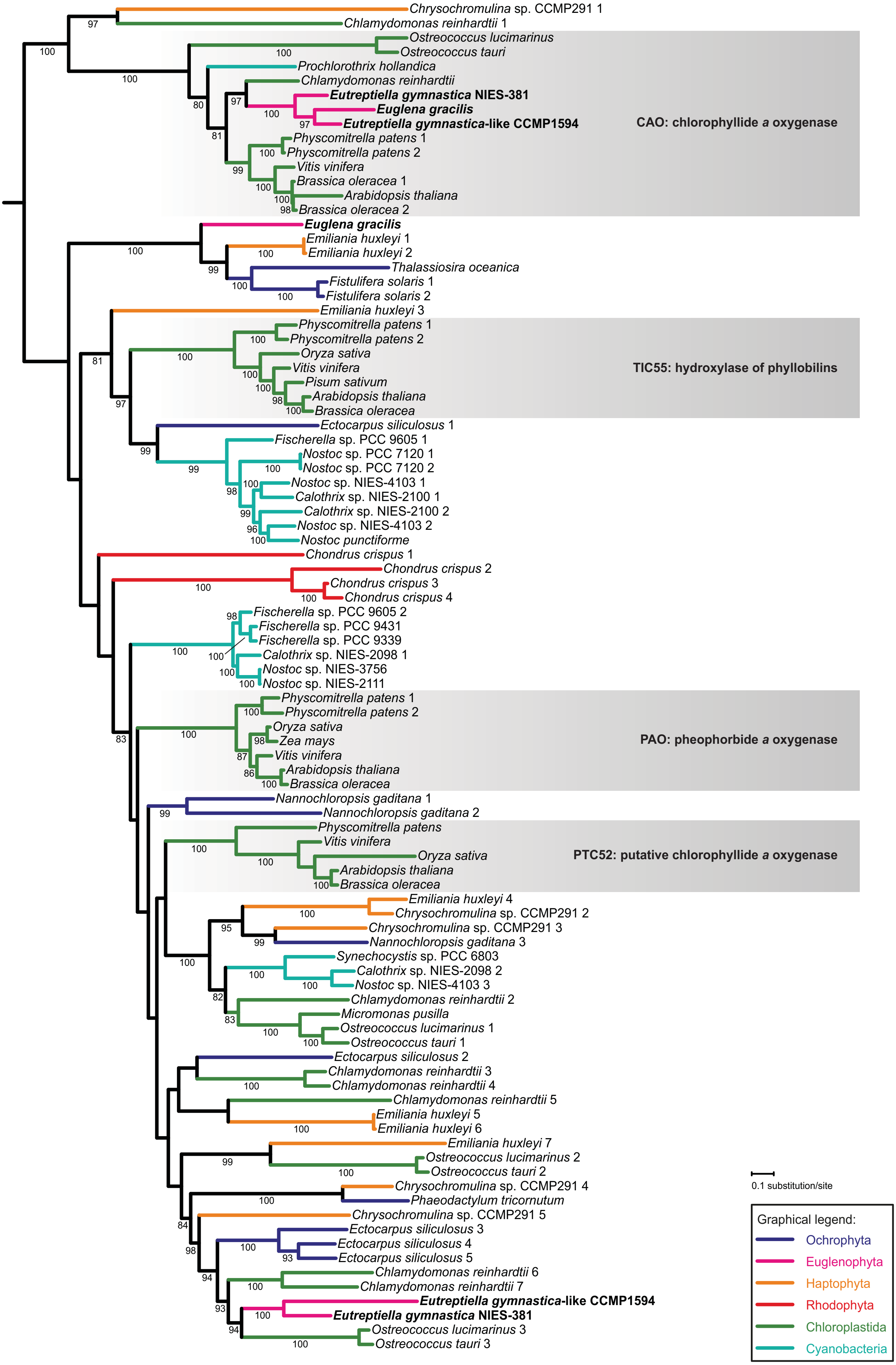

Figure S5: Phylogenetic analysis of Tic55 family proteins. The maximum likelihood phylogenetic tree documents that neither of the euglenophyte Tic55 homologs is a *bona fide* TIC55 protein (grey box named TIC55: hydroxylase of phyllobilins). Three sequences belong to the group of CAO (chlorophyllide *a* oxygenase) proteins, while others represent different branches of the broader family. Bootstrap support values are given when ≥80.

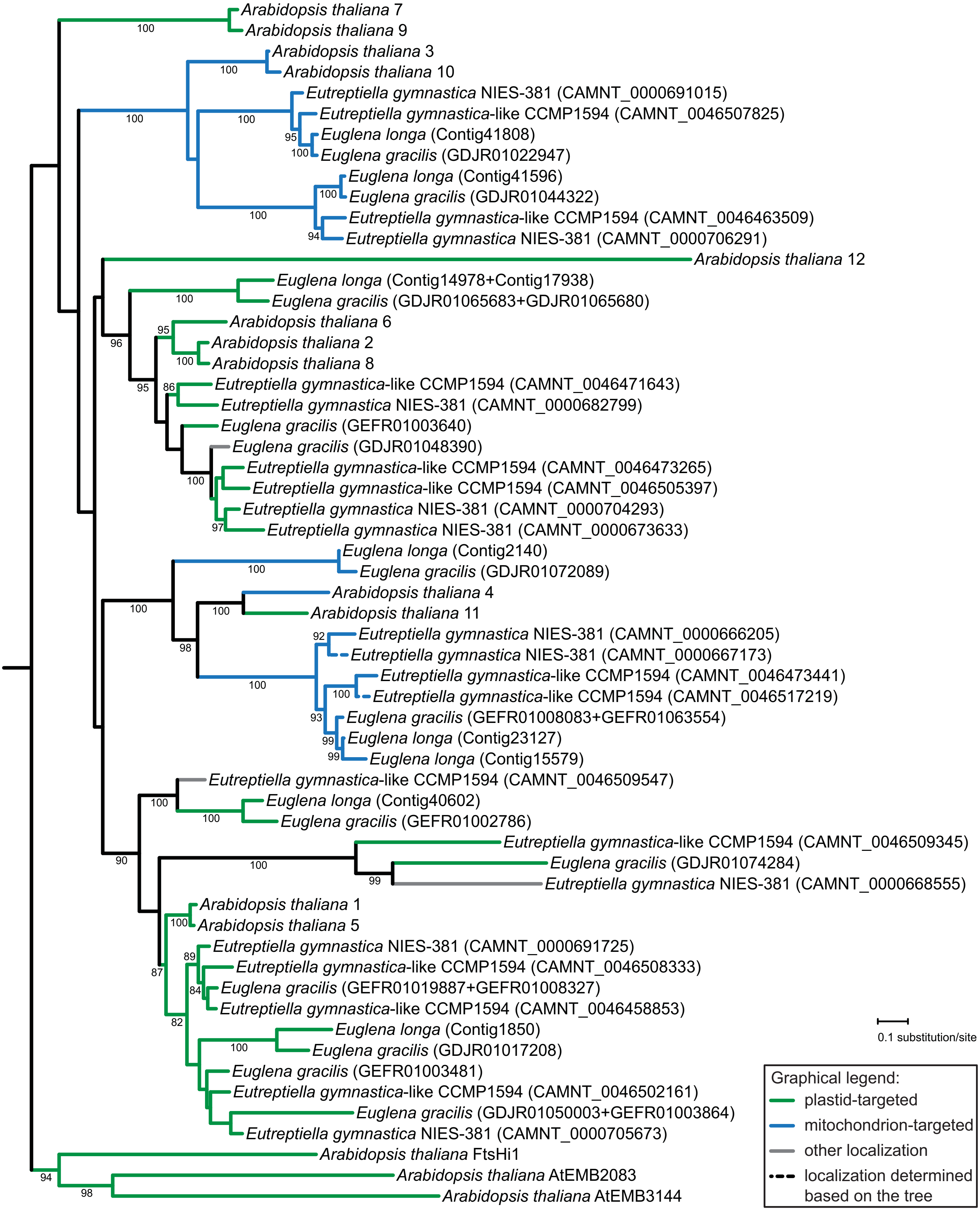

Figure S6: Phylogenetic analysis of FTSH proteases. The maximum likelihood tree was inferred from an alignment of all identified euglenophyte FTSH proteases and the previously described FTSH protease family members from *Arabidopsis thaliana*. Note the reduction of the repertoire of plastid-targeted FTSH proteases in *E. longa* and the absence of euglenophyte orthologs of the *A. thaliana* FTSH12. Dashed lines indicate incomplete sequences whose targeting was indirectly inferred from their position in the tree. Bootstrap support values are given when ≥80.

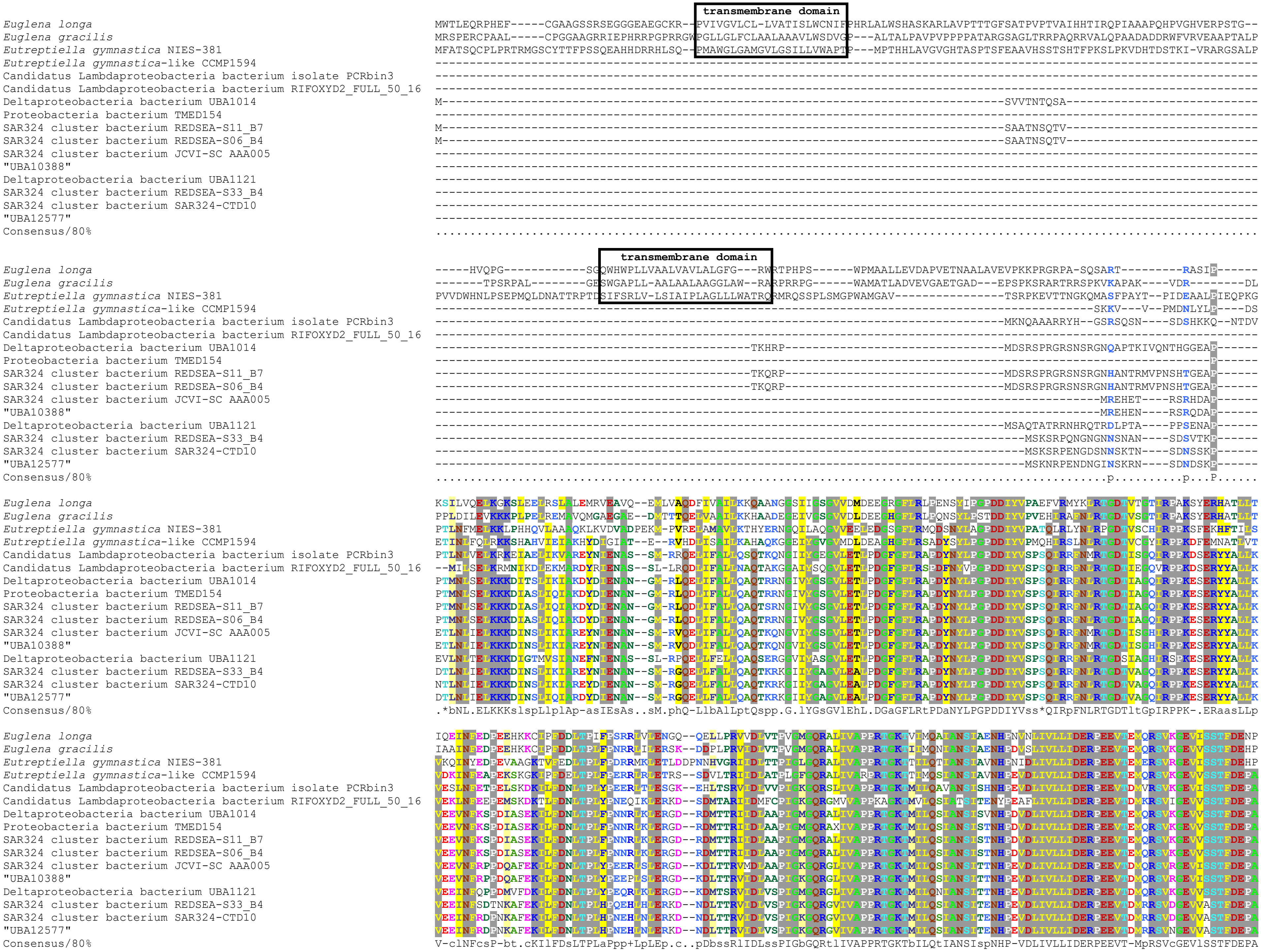
transmembrane domain
transmembrane domain
Euglena longa
Euglena gracilis
Eutreptiella gymnastica NIES-381
Eutreptiella gymnastica-like CCMP1594
Candidatus Lambdaproteobacteria bacterium isolate PCRbin3
Candidatus Lambdaproteobacteria bacterium RIFOXYD2_FULL_50_16
Deltaproteobacteria bacterium UBA1014
Proteobacteria bacterium TMED154
SAR324 cluster bacterium REDSEA-S11_B7
SAR324 cluster bacterium REDSEA-S06_B4
SAR324 cluster bacterium JCVI-SC AAA005
"UBA10388"
Deltaproteobacteria bacterium UBA1121
SAR324 cluster bacterium REDSEA-S33_B4
SAR324 cluster bacterium SAR324-CTD10
"UBA12577"
Consensus/80%

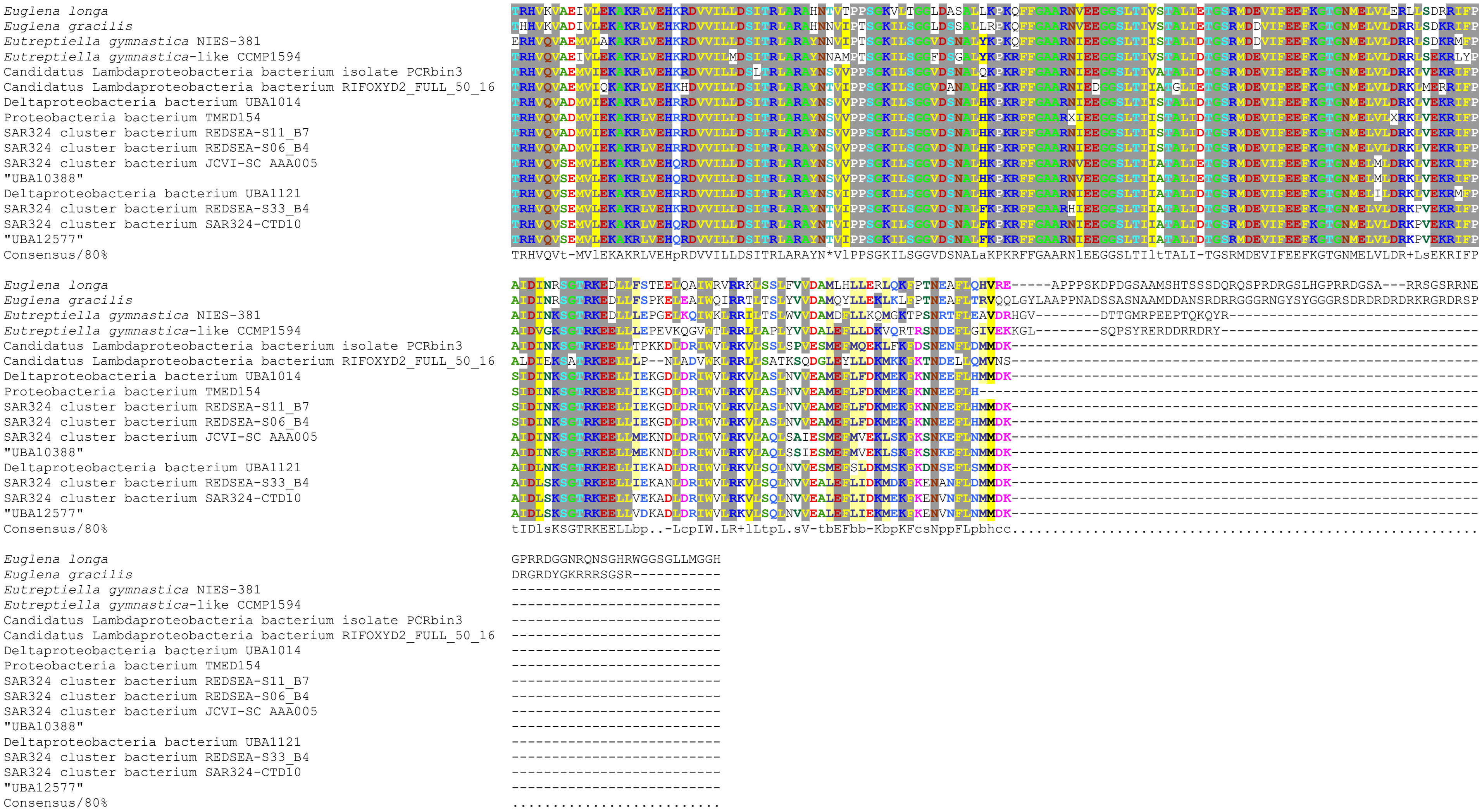

Figure S7: Sequences of the euglenophyte translation-termination factor Rho and their comparison to bacterial homologs. The multiple sequence alignments includes Rho factor sequences from all four euglenophytes analysed and their closest bacterial homologs resolved by a phylogenetic analysis (see Fig. 6). Note the high similarity of the euglenophyte and bacterial sequences, indicative of a conserved function. The euglenophyte sequences exhibit an N-terminal extension bearing all characteristics of a plastid-targeting BTS (note the presence of the two predicted transmembrane domains). The sequence from *Eutreptiella gymnastica*-like CCMP1594 is truncated at the N-terminus.

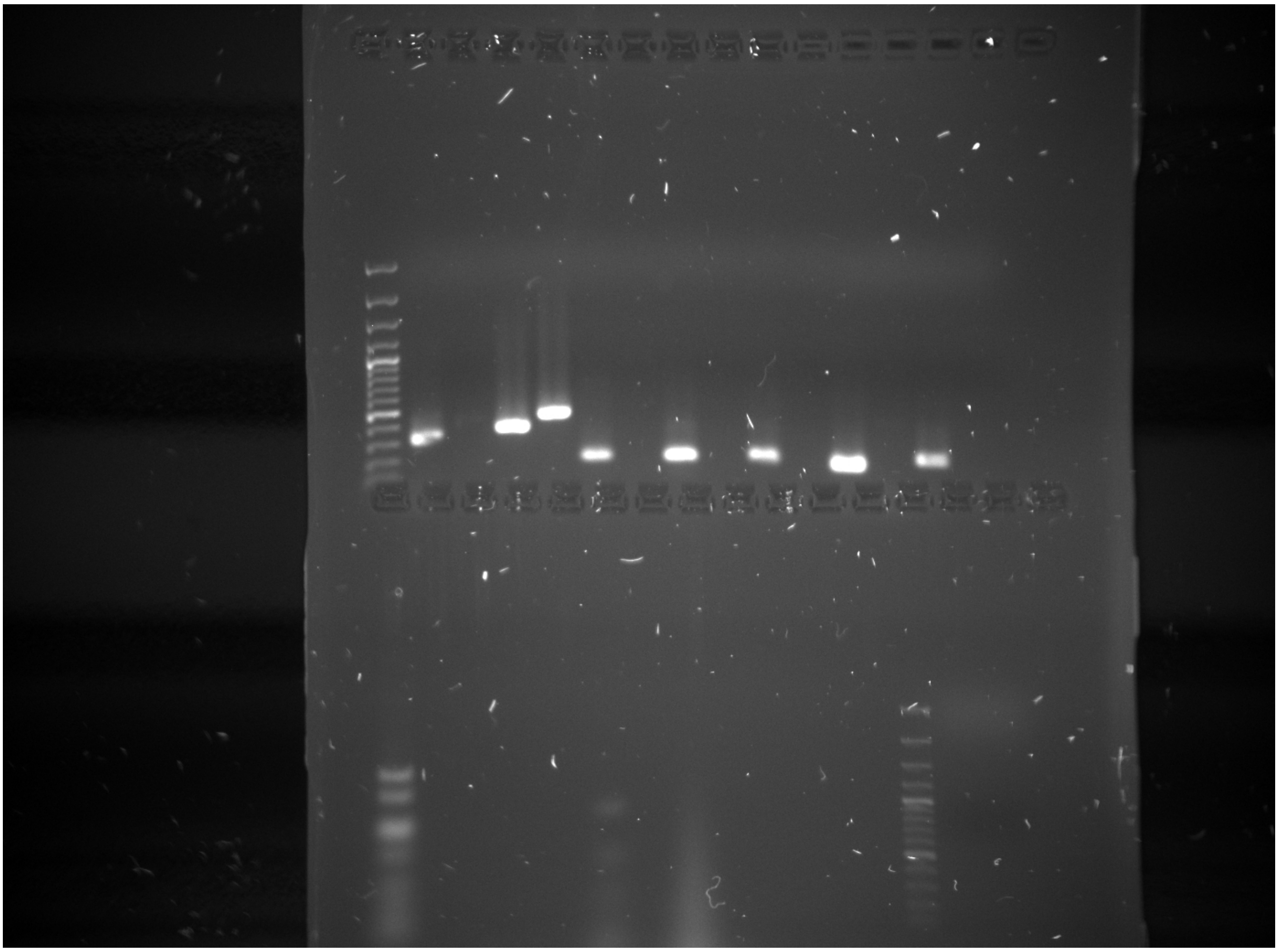

Figure S8: Full original version of the electrophoretic gel, whose part is shown (with an inverted greyscale) in Fig. 1A.

SUPPLEMENTARY DATA S1 - Rho factor full tree Newick

(Euglena longa:0.2454073866,Euglena gracilis:0.1071609403,((Eutreptiella gymnastica-like CCMP1594:0.3737283808,((((OGG94779.1:0.1685048508,Candidatus Lambdaproteobacteria
bacterium RIFOXYC1_FULL_56_13__Candidatus Lambdaproteobacteria_Proteobacteria_Bacteria:0.1008004207)55:0.068073,SAR324 cluster bacterium__SAR324
cluster_Deltaproteobacteria_Proteobacteria_Bacteria:0.1991998136)100:0.296532,((((((SAR324 cluster bacterium SAR324-CTD10 Contig82360
d_Bacteria__p_SAR324__c_SAR324__o_SAR324__f_NAC60-12__g_Arctic96AD-7__s_Arctic96AD-7 sp1:0.0000023338,UBA12577
d_Bacteria__p_SAR324__c_SAR324__o_SAR324__f_NAC60-12__g_Arctic96AD-7__s_Arctic96AD-7 sp2:0.0135532591)100:0.016115,SAR324 cluster bacterium REDSEA-S33_B4 scaffold91
d_Bacteria__p_SAR324__c_SAR324__o_SAR324__f_NAC60-12__g_Arctic96AD-7__s_Arctic96AD-7 sp3:0.0310352670)100:0.175969,Deltaproteobacteria bacterium UBA1121
UBA1121_contig_52842 d_Bacteria__p_SAR324__c_SAR324__o_SAR324__f_NAC60-12__g_UBA8110__s_UBA8110 sp1:0.0842116089)100:0.031041,(SAR324 cluster bacterium JCVI-SC
AAA005 ctg_270 d_Bacteria__p_SAR324__c_SAR324__o_SAR324__f_NAC60-12__g_JCVI-SCAAA005__s_JCVI-SCAAA005 sp1:0.0042855830,UBA10388
d_Bacteria__p_SAR324__c_SAR324__o_SAR324__f_NAC60-12__g_JCVI-SCAAA005__s_JCVI-SCAAA005 sp2:0.0138762315)100:0.112689)99:0.038696,(Deltaproteobacteria bacterium
UBA1014 UBA1014_contig_2062 d_Bacteria__p_SAR324__c_SAR324__o_SAR324__f_NAC60-12__g_UBA1014__s_UBA1014 sp1:0.0045134975,((SAR324 cluster bacterium REDSEA-
S11_B7 scaffold89 d_Bacteria__p_SAR324__c_SAR324__o_SAR324__f_NAC60-12__g_UBA1014__s_UBA1014 sp2:0.0000023338,SAR324 cluster bacterium REDSEA-S06_B4 scaffold119
GTDB Taxonomy_Undefined _Failed Quality Check_ 1 mismatch from s_UBA1014 sp2:0.0000023338)69:0.000003,Proteobacteria bacterium
TMED154__Proteobacteria_Bacteria:0.0000023338)99:0.004285)99:0.128756)31:0.039085,Candidatus Lambdaproteobacteria bacterium isolate PCRbin3
scaffold145:0.1944772611)32:0.034464)19:0.076709,(((Geothermobacter sp. HR-
1__Geothermobacter_Geobacteraceae_Desulfuromonadales_Deltaproteobacteria_Proteobacteria_Bacteria:0.0921128103,(Geoalkalibacter
ferrihydriticus__Geoalkalibacter_Geobacteraceae_Desulfuromonadales_Deltaproteobacteria_Proteobacteria_Bacteria:0.0919029214,(Desulfuromonas acetoxidans DSM
684__Desulfuromonas_Desulfuromonadaceae_Desulfuromonadales_Deltaproteobacteria_Proteobacteria_Bacteria:0.1359627243,Deltaproteobacteria bacterium HGW-
Deltaproteobacteria-4__Deltaproteobacteria_Proteobacteria_Bacteria:0.0866248835)98:0.021889)98:0.022295)58:0.010264,((((Pelobacter
acetylenicus__Pelobacter_Desulfuromonadaceae_Desulfuromonadales_Deltaproteobacteria_Proteobacteria_Bacteria:0.0843323056,Pelobacter sp.
SFB93__Pelobacter_Desulfuromonadaceae_Desulfuromonadales_Deltaproteobacteria_Proteobacteria_Bacteria:0.0444400910)98:0.033698,((Desulfuromonadales bacterium
C00003093__Desulfuromonadales_Deltaproteobacteria_Proteobacteria_Bacteria:0.0994831335,Desulfuromusa
kysingii__Desulfuromusa_Desulfuromonadaceae_Desulfuromonadales_Deltaproteobacteria_Proteobacteria_Bacteria:0.1113619984)100:0.029728,Geopsychrobacter
electrodiphilus__Geopsychrobacter_Geobacteraceae_Desulfuromonadales_Deltaproteobacteria_Proteobacteria_Bacteria:0.1461550384)100:0.100917)42:0.002964,Desulfuromonas
sp.__Desulfuromonas_Desulfuromonadaceae_Desulfuromonadales_Deltaproteobacteria_Proteobacteria_Bacteria:0.0715751841)43:0.012854,(((Desulfuromonas
sp.__Desulfuromonas_Desulfuromonadaceae_Desulfuromonadales_Deltaproteobacteria_Proteobacteria_Bacteria:0.0494176536,Desulfuromonas sp.
DDH964__Desulfuromonas_Desulfuromonadaceae_Desulfuromonadales_Deltaproteobacteria_Proteobacteria_Bacteria:0.0257743651)99:0.020152,Desulfuromonas
sp.__Desulfuromonas_Desulfuromonadaceae_Desulfuromonadales_Deltaproteobacteria_Proteobacteria_Bacteria:0.0367949605)98:0.013216,Desulfuromonas
sp.__Desulfuromonas_Desulfuromonadaceae_Desulfuromonadales_Deltaproteobacteria_Proteobacteria_Bacteria:0.0703874259)98:0.028793)32:0.010314)100:0.125524,(((((((((((((Ga
mmaproteobacteria bacterium__Gammaproteobacteria_Proteobacteria_Bacteria:0.0986601772,Methylophaga
aminisulfidivorans__Methylophaga_Piscirickettsiaceae_Thiotrichales_Gammaproteobacteria_Proteobacteria_Bacteria:0.1165975437)100:0.266530,(((((Gammaproteobacteria bacterium
SG8_15__Gammaproteobacteria_Proteobacteria_Bacteria:0.1138794890,((((((Algicola
sagamiensis__Algicola_Pseudoalteromonadaceae_Alteromonadales_Gammaproteobacteria_Proteobacteria_Bacteria:0.0452406947,Pseudoalteromonas
ulvae__Pseudoalteromonas_Pseudoalteromonadaceae_Alteromonadales_Gammaproteobacteria_Proteobacteria_Bacteria:0.0871108950)100:0.043128,Ferrimonas
balearica__Ferrimonas_Ferrimonadaceae_Alteromonadales_Gammaproteobacteria_Proteobacteria_Bacteria:0.0775566822)100:0.019877,Idiomarina
xiamenensis__Idiomarina_Idiomarinaceae_Alteromonadales_Gammaproteobacteria_Proteobacteria_Bacteria:0.0741299547)100:0.140580,((Endozoicomonas
elysicola__Endozoicomonas_Hahellaceae_Oceanospirillales_Gammaproteobacteria_Proteobacteria_Bacteria:0.0817556924,(Motiliproteus sp. MSK22-
1__Motiliproteus_Oceanospirillales_Gammaproteobacteria_Proteobacteria_Bacteria:0.1197722849,(Thalassolituus sp.
CG17_big_fil_post_rev_8_21_14_2_50_53_8__Thalassolituus_Oceanospirillales_Gammaproteobacteria_Proteobacteria_Bacteria:0.0691289361,Bacterioplanes
sanyensis__Bacterioplanes_Oceanospirillales_Gammaproteobacteria_Proteobacteria_Bacteria:0.0616847998)100:0.185081)78:0.082321)100:0.069361,Legionella
sp.__Legionella_Legionellaceae_Legionellales_Gammaproteobacteria_Proteobacteria_Bacteria:0.3179032408)95:0.025615)81:0.022432,Coxiella sp.
DG_40__Coxiella_Coxiellaceae_Legionellales_Gammaproteobacteria_Proteobacteria_Bacteria:0.1891986449)94:0.057402,Gammaproteobacteria bacterium
TMED36__Gammaproteobacteria_Proteobacteria_Bacteria:0.4180340603)62:0.016645)71:0.014564,Candidatus Tenderia electrophaga__Candidatus
Tenderia_Chromatiaceae_Chromatiales_Gammaproteobacteria_Proteobacteria_Bacteria:0.1408187123)76:0.019681,(bacterium BMS3Bbin12 Bacteria.__:0.1342390854,Candidatus
Competibacter denitrificans__Candidatus Competibacter_Competibacteraceae_Gammaproteobacteria_Proteobacteria_Bacteria:0.1394531005)94:0.036123)87:0.028733,Candidatus
Thiomargarita nelsonii__Thiomargarita_Thiotrichaceae_Thiotrichales_Gammaproteobacteria_Proteobacteria_Bacteria:0.2386490942)87:0.025091,Nitrosococcus
watsonii__Nitrosococcus_Chromatiaceae_Chromatiales_Gammaproteobacteria_Proteobacteria_Bacteria:0.1648962600)87:0.033480)89:0.050122,Acidithiobacillales bacterium
SG8_45__Acidithiobacillales_Acidithiobacillia_Proteobacteria_Bacteria:0.2580088435)97:0.068953,(Thermithiobacillus
tepidarius__Thermithiobacillus_Thermithiobacillaceae_Acidithiobacillales_Acidithiobacillia_Proteobacteria_Bacteria:0.0477045728,(Acidithiobacillus caldus SM-
1__Acidithiobacillus_Acidithiobacillaceae_Acidithiobacillales_Acidithiobacillia_Proteobacteria_Bacteria:0.0259065974,Acidithiobacillus ferrivorans
SS3__Acidithiobacillus_Acidithiobacillaceae_Acidithiobacillales_Acidithiobacillia_Proteobacteria_Bacteria:0.0423585202)100:0.149970)100:0.116524)100:0.147834,(((((((((((Candidatus
Puniceispirillum marinum IMCC1322__Candidatus Puniceispirillum_SAR116 cluster_Alphaproteobacteria_Proteobacteria_Bacteria:0.0498443321,Candidatus Puniceispirillum sp.
TMED52__Candidatus Puniceispirillum_SAR116 cluster_Alphaproteobacteria_Proteobacteria_Bacteria:0.1551220806)100:0.100896,Candidatus Endolissoclinum sp.
TMED26__Candidatus Endolissoclinum_Rhodospirillaceae_Rhodospirillales_Alphaproteobacteria_Proteobacteria_Bacteria:0.1120899188)66:0.037005,((Oceanibaculum
nanhaiense__Oceanibaculum_Rhodospirillaceae_Rhodospirillales_Alphaproteobacteria_Proteobacteria_Bacteria:0.0344470375,Oceanibaculum
pacificum__Oceanibaculum_Rhodospirillaceae_Rhodospirillales_Alphaproteobacteria_Proteobacteria_Bacteria:0.0121975962)100:0.070893,Magnetospirillum gryphiswaldense MSR-
1__Magnetospirillum_Rhodospirillaceae_Rhodospirillales_Alphaproteobacteria_Proteobacteria_Bacteria:0.1001418173)52:0.013088)85:0.016306,(Rhodospirillales bacterium
47_12_T64__unclassified Rhodospirillales_Rhodospirillales_Alphaproteobacteria_Proteobacteria_Bacteria:0.1167871838,Fodinicurvata
fenggangensis__Fodinicurvata_Rhodospirillaceae_Rhodospirillales_Alphaproteobacteria_Proteobacteria_Bacteria:0.1467474263)78:0.071493)57:0.025346,(((Rhodospirillales bacterium
20-60-12__unclassified Rhodospirillales_Rhodospirillales_Alphaproteobacteria_Proteobacteria_Bacteria:0.0588527495,(((Acidocella
aminolytica__Acidocella_Acetobacteraceae_Rhodospirillales_Alphaproteobacteria_Proteobacteria_Bacteria:0.0162773844,Acidocella__Acetobacteraceae_Rhodospirillales_Alphaproteo
bacteria_Proteobacteria_Bacteria:0.0207487411)100:0.097883,Rhodospirillales bacterium 20-64-7__unclassified
Rhodospirillales_Rhodospirillales_Alphaproteobacteria_Proteobacteria_Bacteria:0.0358830522)100:0.051927,(Acidiphilium sp. 21-60-
14__Acidiphilium_Acetobacteraceae_Rhodospirillales_Alphaproteobacteria_Proteobacteria_Bacteria:0.0382987101,Acidiphilium__Acetobacteraceae_Rhodospirillales_Alphaproteobact
eria_Proteobacteria_Bacteria:0.0387232149)100:0.061550)100:0.038417)100:0.038632,((((Neoasaia
chiangmaiensis__Neoasaia_Acetobacteraceae_Rhodospirillales_Alphaproteobacteria_Proteobacteria_Bacteria:0.0044869253,Gluconobacter morbifer
G707__Gluconobacter_Acetobacteraceae_Rhodospirillales_Alphaproteobacteria_Proteobacteria_Bacteria:0.0521219162)98:0.009084,(Kozakia
baliensis__Kozakia_Acetobacteraceae_Rhodospirillales_Alphaproteobacteria_Proteobacteria_Bacteria:0.0178704978,Tanticharoenia
sakaeratensis__Tanticharoenia_Acetobacteraceae_Rhodospirillales_Alphaproteobacteria_Proteobacteria_Bacteria:0.0534773389)86:0.000002)100:0.033190,((((((Acetobacter
syzygii__Acetobacter_Acetobacteraceae_Rhodospirillales_Alphaproteobacteria_Proteobacteria_Bacteria:0.0000000000,Acetobacter
okinawensis__Acetobacter_Acetobacteraceae_Rhodospirillales_Alphaproteobacteria_Proteobacteria_Bacteria:0.0000000000):0.000000,Acetobacter__Acetobacteraceae_Rhodospirillale
s_Alphaproteobacteria_Proteobacteria_Bacteria:0.0000000000):0.000000,Acetobacter
okinawensis__Acetobacter_Acetobacteraceae_Rhodospirillales_Alphaproteobacteria_Proteobacteria_Bacteria:0.0000000000):0.000002,Acetobacter
ghanensis__Acetobacter_Acetobacteraceae_Rhodospirillales_Alphaproteobacteria_Proteobacteria_Bacteria:0.0000023226)100:0.009000,Acetobacter
pasteurianus__Acetobacter_Acetobacteraceae_Rhodospirillales_Alphaproteobacteria_Proteobacteria_Bacteria:0.0000023873)100:0.038796,Acetobacter__Acetobacteraceae_Rhodospir
illales_Alphaproteobacteria_Proteobacteria_Bacteria:0.0370842235)100:0.025413,(Acetobacter aceti__Acetobacter subgen.
Acetobacter_Acetobacter_Acetobacteraceae_Rhodospirillales_Alphaproteobacteria_Proteobacteria_Bacteria:0.0386657182,Acetobacter
nitrogenifigens__Acetobacter_Acetobacteraceae_Rhodospirillales_Alphaproteobacteria_Proteobacteria_Bacteria:0.0230264661)100:0.017113)99:0.010994)99:0.016672,(Gluconacetoba
cter diazotrophicus__Gluconacetobacter_Acetobacteraceae_Rhodospirillales_Alphaproteobacteria_Proteobacteria_Bacteria:0.0146084425,Komagataeibacter
hansenii__Komagataeibacter_Acetobacteraceae_Rhodospirillales_Alphaproteobacteria_Proteobacteria_Bacteria:0.0581890264)86:0.009187)100:0.068339)98:0.031623,Elioraea
tepidiphila__Elioraea_unclassified Rhodospirillales_Rhodospirillales_Alphaproteobacteria_Proteobacteria_Bacteria:0.1303361735)99:0.056071)40:0.005224,(((((Alphaproteobacteria

bacterium GWF2_58_20__Alphaproteobacteria_Proteobacteria_Bacteria:0.2246958872,Tistrella
mobilis__Tistrella_Rhodospirillaceae_Rhodospirillales_Alphaproteobacteria_Proteobacteria_Bacteria:0.2112129639)73:0.038188,(Niveispirillum
irakense__Niveispirillum_Rhodospirillaceae_Rhodospirillales_Alphaproteobacteria_Proteobacteria_Bacteria:0.1242406603,(Azospirillum
brasilense__Azospirillum_Rhodospirillaceae_Rhodospirillales_Alphaproteobacteria_Proteobacteria_Bacteria:0.0171597909,Azospirillum sp.
B510__Azospirillum_Rhodospirillaceae_Rhodospirillales_Alphaproteobacteria_Proteobacteria_Bacteria:0.0365239112)100:0.070888)92:0.037312)64:0.024839,(((uncultured
Alphaproteobacteria bacterium__environmental samples_Alphaproteobacteria_Proteobacteria_Bacteria:0.0940811313,(Rhodospira
trueperi__Rhodospira_Rhodospirillaceae_Rhodospirillales_Alphaproteobacteria_Proteobacteria_Bacteria:0.0000023338,Rhodospira
trueperi__Rhodospira_Rhodospirillaceae_Rhodospirillales_Alphaproteobacteria_Proteobacteria_Bacteria:0.0000023338)100:0.140556)70:0.024915,(Roseospirillum
parvum__Roseospirillum_Rhodobiaceae_Rhizobiales_Alphaproteobacteria_Proteobacteria_Bacteria:0.1209480895,(Caedibacter sp. 37-
49__Caedibacter_Holosporales_Alphaproteobacteria_Proteobacteria_Bacteria:0.1791261950,Caedimonas
varicaedens__Caedimonas_Caedimonadaceae_Holosporales_Alphaproteobacteria_Proteobacteria_Bacteria:0.1461191493)100:0.086544)82:0.030803)80:0.008090,Novispirillum
itersonii__Novispirillum_Rhodospirillaceae_Rhodospirillales_Alphaproteobacteria_Proteobacteria_Bacteria:0.1587071602)74:0.012414)56:0.010706,Rhodospirillaceae
bacterium__Rhodospirillaceae_Rhodospirillales_Alphaproteobacteria_Proteobacteria_Bacteria:0.2128820915)52:0.004947,Alphaproteobacteria bacterium 41-
28__Alphaproteobacteria_Proteobacteria_Bacteria:0.2403200494)47:0.011673)86:0.027436,(Candidatus Endolissoclinum sp. TMED37__Candidatus
Endolissoclinum_Rhodospirillaceae_Rhodospirillales_Alphaproteobacteria_Proteobacteria_Bacteria:0.1845069516,((Rhodospirillaceae bacterium
TMED167__Rhodospirillaceae_Rhodospirillales_Alphaproteobacteria_Proteobacteria_Bacteria:0.1406525887,Rhodospirillales bacterium RIFCSPLOWO2_02_FULL_58_16__unclassified
Rhodospirillales_Rhodospirillales_Alphaproteobacteria_Proteobacteria_Bacteria:0.1441072809)96:0.038206,Terasakiella sp.
PR1__Terasakiella_Methylocystaceae_Rhizobiales_Alphaproteobacteria_Proteobacteria_Bacteria:0.1643566526)92:0.022754)84:0.008095)86:0.019541,(Candidatus Odyssella
thessalonicensis__Candidatus Odyssella_Candidatus Paracaedibacteraceae_Holosporales_Alphaproteobacteria_Proteobacteria_Bacteria:0.0316406648,Candidatus Paracaedibacter
acanthamoebae__Candidatus Paracaedibacter_Candidatus
Paracaedibacteraceae_Holosporales_Alphaproteobacteria_Proteobacteria_Bacteria:0.0298210916)100:0.154240)76:0.014297,(Alphaproteobacteria bacterium
CG11_big_fil_rev_8_21_14_0_20_39_49__Alphaproteobacteria_Proteobacteria_Bacteria:0.3295106307,Alphaproteobacteria
bacterium__Alphaproteobacteria_Proteobacteria_Bacteria:0.2648058121)89:0.062353)90:0.045239,((((((((Xanthobacter
autotrophicus__Xanthobacter_Xanthobacteraceae_Rhizobiales_Alphaproteobacteria_Proteobacteria_Bacteria:0.0350845326,(Rhizobiales bacterium 39-66-
18__Rhizobiales_Alphaproteobacteria_Proteobacteria_Bacteria:0.0138435142,(Rhizobiales bacterium 17-65-
6__Rhizobiales_Alphaproteobacteria_Proteobacteria_Bacteria:0.0000023229,Rhizobiales bacterium 12-68-
15__Rhizobiales_Alphaproteobacteria_Proteobacteria_Bacteria:0.0045683792)100:0.022777)100:0.014069)99:0.019688,(Bosea sp.
117__Bosea_Bradyrhizobiaceae_Rhizobiales_Alphaproteobacteria_Proteobacteria_Bacteria:0.0088674355,Ancylobacter__Xanthobacteraceae_Rhizobiales_Alphaproteobacteria_Proteo
bacteria_Bacteria:0.0387871387)100:0.029007)81:0.013376,(((unclassified Rhizobiales
miscellaneous__Rhizobiales_Alphaproteobacteria_Proteobacteria_Bacteria:0.0631798976,Rhizobiales bacterium 65-
9__Rhizobiales_Alphaproteobacteria_Proteobacteria_Bacteria:0.1195467254)72:0.015637,((Chelatococcus__Chelatococcaceae_Rhizobiales_Alphaproteobacteria_Proteobacteria_Bacter
ia:0.0089305922,Chelatococcus__Chelatococcaceae_Rhizobiales_Alphaproteobacteria_Proteobacteria_Bacteria:0.0000024009)100:0.015425,(Microvirga sp.
BSC39__Microvirga_Methylobacteriaceae_Rhizobiales_Alphaproteobacteria_Proteobacteria_Bacteria:0.0467770492,(Methylobacterium extorquens
PA1__Methylobacterium_Methylobacteriaceae_Rhizobiales_Alphaproteobacteria_Proteobacteria_Bacteria:0.0139693550,Methylobacterium sp. ARG-
1__Methylobacterium_Methylobacteriaceae_Rhizobiales_Alphaproteobacteria_Proteobacteria_Bacteria:0.0188343604)100:0.090373)100:0.057546)49:0.016317)44:0.012631,(Labrys
sp. WJW__Labrys_Xanthobacteraceae_Rhizobiales_Alphaproteobacteria_Proteobacteria_Bacteria:0.0966241714,Bosea sp.
AAP35__Bosea_Bradyrhizobiaceae_Rhizobiales_Alphaproteobacteria_Proteobacteria_Bacteria:0.1275957014)37:0.016184)51:0.007788)97:0.031464,((((Pseudoxanthobacter
soli__Pseudoxanthobacter_Xanthobacteraceae_Rhizobiales_Alphaproteobacteria_Proteobacteria_Bacteria:0.0481594487,((Amorphus
coralli__Amorphus_Rhodobiaceae_Rhizobiales_Alphaproteobacteria_Proteobacteria_Bacteria:0.0573703605,(Labrenzia sp.
OB1__Labrenzia_Rhodobacteraceae_Rhodobacterales_Alphaproteobacteria_Proteobacteria_Bacteria:0.0715041443,Roseibium sp.
TrichSKD4__Roseibium_Rhodobacteraceae_Rhodobacterales_Alphaproteobacteria_Proteobacteria_Bacteria:0.0325397934)100:0.036286)85:0.013999,Pleomorphomonas
oryzae__Pleomorphomonas_Methylocystaceae_Rhizobiales_Alphaproteobacteria_Proteobacteria_Bacteria:0.0788598110)86:0.022115)86:0.012155,Kaistia
adipata__Kaistia_Rhizobiaceae_Rhizobiales_Alphaproteobacteria_Proteobacteria_Bacteria:0.1123501610)72:0.026856,((((Hyphomicrobium sp.
CS1BSMeth3__Hyphomicrobium_Hyphomicrobiaceae_Rhizobiales_Alphaproteobacteria_Proteobacteria_Bacteria:0.0574145180,Hyphomicrobium sp. 12-62-
95__Hyphomicrobium_Hyphomicrobiaceae_Rhizobiales_Alphaproteobacteria_Proteobacteria_Bacteria:0.0707576194)100:0.011555,Filomicrobium__Hyphomicrobiaceae_Rhizobiales_A
lphaproteobacteria_Proteobacteria_Bacteria:0.0420769487)100:0.026890,Hyphomicrobium
nitrativorans__Hyphomicrobium_Hyphomicrobiaceae_Rhizobiales_Alphaproteobacteria_Proteobacteria_Bacteria:0.0297195832)100:0.130202,((Methylopila sp.
M107__Methylopila_Methylocystaceae_Rhizobiales_Alphaproteobacteria_Proteobacteria_Bacteria:0.0229072534,Methylopila sp.
Yamaguchi__Methylopila_Methylocystaceae_Rhizobiales_Alphaproteobacteria_Proteobacteria_Bacteria:0.0127006852)100:0.082743,((((Thermopetrobacter sp.
TC1__Thermopetrobacter_Alphaproteobacteria_Proteobacteria_Bacteria:0.1635864021,Methyloceanibacter
caenitepidi__Methyloceanibacter_Rhizobiales_Alphaproteobacteria_Proteobacteria_Bacteria:0.0844607735)83:0.020111,(alpha proteobacterium Mf
1.05b.01__Alphaproteobacteria_Proteobacteria_Bacteria:0.0994613699,((Chelativorans sp.
BNC1__Chelativorans_Phyllobacteriaceae_Rhizobiales_Alphaproteobacteria_Proteobacteria_Bacteria:0.0391582233,Bartonella
ancashensis__Bartonella_Bartonellaceae_Rhizobiales_Alphaproteobacteria_Proteobacteria_Bacteria:0.1663572522)98:0.016448,Rhizobiales
bacterium__Rhizobiales_Alphaproteobacteria_Proteobacteria_Bacteria:0.1088843830)100:0.091151)98:0.025898)75:0.016614,Lutibaculum
baratangense__Lutibaculum_Rhodobiaceae_Rhizobiales_Alphaproteobacteria_Proteobacteria_Bacteria:0.1277504102)63:0.010540,Rhizobiales
bacterium__Rhizobiales_Alphaproteobacteria_Proteobacteria_Bacteria:0.1223725732)68:0.021961)45:0.006458)43:0.002938)53:0.005772,(alpha proteobacterium
IMCC14465__Alphaproteobacteria_Proteobacteria_Bacteria:0.1678268706,((Pelagibacterium__Hyphomicrobiaceae_Rhizobiales_Alphaproteobacteria_Proteobacteria_Bacteria:0.04279
53452,Maritalea myrionectae__Maritalea_Hyphomicrobiaceae_Rhizobiales_Alphaproteobacteria_Proteobacteria_Bacteria:0.0816329179)99:0.028903,Cucumibacter
marinus__Cucumibacter_Hyphomicrobiaceae_Rhizobiales_Alphaproteobacteria_Proteobacteria_Bacteria:0.0318978221)100:0.026765)92:0.045451)57:0.010530)67:0.026029,Rhodopla
nes sp. Z2-YC6860__Rhodoplanes_Hyphomicrobiaceae_Rhizobiales_Alphaproteobacteria_Proteobacteria_Bacteria:0.0893523308)84:0.053634,(Euryhalocaulis
caribicus__Euryhalocaulis_Hyphomonadaceae_Rhodobacterales_Alphaproteobacteria_Proteobacteria_Bacteria:0.1862010712,(((Phaeobacter gallaeciensis DSM
26640__Phaeobacter_Rhodobacteraceae_Rhodobacterales_Alphaproteobacteria_Proteobacteria_Bacteria:0.0446750251,Rhodobacteraceae
bacterium__Rhodobacteraceae_Rhodobacterales_Alphaproteobacteria_Proteobacteria_Bacteria:0.1228101965)100:0.054753,Natronohydrobacter
thiooxidans__Natronohydrobacter_Rhodobacteraceae_Rhodobacterales_Alphaproteobacteria_Proteobacteria_Bacteria:0.1218103360)100:0.115785,Henriciella
marina__Henriciella_Hyphomonadaceae_Rhodobacterales_Alphaproteobacteria_Proteobacteria_Bacteria:0.2480282869)98:0.067780)96:0.027087)100:0.034297,Candidatus
Fonsibacter ubiquis__Candidatus Fonsibacter_Pelagibacterales_Alphaproteobacteria_Proteobacteria_Bacteria:0.3332872955)95:0.043206,(alpha proteobacterium Q-
1__Alphaproteobacteria_Proteobacteria_Bacteria:0.2039494600,(Kordiimonas
lipolytica__Kordiimonas_Kordiimonadaceae_Kordiimonadales_Alphaproteobacteria_Proteobacteria_Bacteria:0.0589549690,Kordiimonadales bacterium__unclassified
Kordiimonadales_Kordiimonadales_Alphaproteobacteria_Proteobacteria_Bacteria:0.1202066705)100:0.044735)99:0.095697)40:0.010808)74:0.034023,Sneathiella
glossodoripedis__Sneathiella_Sneathiellaceae_Sneathiellales_Alphaproteobacteria_Proteobacteria_Bacteria:0.1477933350)100:0.134104,Magnetococcus marinus MC-
1__Magnetococcus_Magnetococcaceae_Magnetococcales_Alphaproteobacteria_Proteobacteria_Bacteria:0.3441493265)97:0.063271)100:0.113416,(Desulfocurvus
vexinensis__Desulfocurvus_Desulfovibrionaceae_Desulfovibrionales_Deltaproteobacteria_Proteobacteria_Bacteria:0.1462724611,((((Desulfovibrio vulgaris
DP4__Desulfovibrio_Desulfovibrionaceae_Desulfovibrionales_Deltaproteobacteria_Proteobacteria_Bacteria:0.0333687768,uncultured delta proteobacterium__environmental
samples_Deltaproteobacteria_Proteobacteria_Bacteria:0.1004683414)89:0.024185,Desulfovibrio alaskensis
G20__Desulfovibrio_Desulfovibrionaceae_Desulfovibrionales_Deltaproteobacteria_Proteobacteria_Bacteria:0.0680554098)100:0.053844,Desulfovibrio
gigas__Desulfovibrio_Desulfovibrionaceae_Desulfovibrionales_Deltaproteobacteria_Proteobacteria_Bacteria:0.1546105983)100:0.085566,Pseudodesulfovibrio
piezophilus__Desulfovibrionaceae_Desulfovibrionales_Deltaproteobacteria_Proteobacteria_Bacteria:0.1277208097)98:0.024508)100:0.233356)86:0.029152,Calditerrivibrio
nitroreducens__Deferribacteraceae_Deferribacterales_Deferribacteres_Bacteria:0.4437533724)80:0.035880,(((Deltaproteobacteria
bacterium__Deltaproteobacteria_Proteobacteria_Bacteria:0.2665441810,Deferrisoma
camini__Deferrisoma_Deltaproteobacteria_Proteobacteria_Bacteria:0.1300821288)100:0.170451,((bacterium TMED88 Bacteria.__:0.0980147109,Opitutales bacterium
TMED158__Opitutales_Opitutae_Verrucomicrobia_Bacteria:0.1261885810)100:0.316931,Deltaproteobacteria bacterium
CG11_big_fil_rev_8_21_14_0_20_45_16__Deltaproteobacteria_Proteobacteria_Bacteria:0.3174104369)79:0.034986)87:0.035110,Thermodesulfatator
autotrophicus__Thermodesulfatator_Thermodesulfobacteriaceae_Thermodesulfobacteriales_Thermodesulfobacteria_Bacteria:0.2950632627)79:0.009288)88:0.042923,(Desulfuromona
dales bacterium C00003096__Desulfuromonadales_Deltaproteobacteria_Proteobacteria_Bacteria:0.0507118991,delta proteobacterium
ML8_D__Deltaproteobacteria_Proteobacteria_Bacteria:0.0681258153)100:0.149495)85:0.020354,(((((((((Bdellovibrio sp.
ArHS__Bdellovibrio_Bdellovibrionaceae_Bdellovibrionales_Oligoflexia_Proteobacteria_Bacteria:0.0000024991,Bdellovibrio
bacteriovorus__Bdellovibrio_Bdellovibrionaceae_Bdellovibrionales_Oligoflexia_Proteobacteria_Bacteria:0.0045387848)94:0.011123,Bdellovibrio

bacteriovorus__Bdellovibrio_Bdellovibrionaceae_Bdellovibrionales_Oligoflexia_Proteobacteria_Bacteria:0.0219394113)87:0.000002,Bdellovibrio sp.
SKB1291214__Bdellovibrio_Bdellovibrionaceae_Bdellovibrionales_Oligoflexia_Proteobacteria_Bacteria:0.0496003256)91:0.030350,(Bdellovibrionales bacterium
RIFCSPHIGHO2_01_FULL_40_29__Bdellovibrionales_Oligoflexia_Proteobacteria_Bacteria:0.0456552348,Bdellovibrio
exovorus__Bdellovibrio_Bdellovibrionaceae_Bdellovibrionales_Oligoflexia_Proteobacteria_Bacteria:0.0236143380)100:0.102753)98:0.053656,Bdellovibrionales bacterium
RBG_16_40_8__Bdellovibrionales_Oligoflexia_Proteobacteria_Bacteria:0.0918313466)81:0.057989,Proteobacteria bacterium
SG_bin7__Proteobacteria_Bacteria:0.1243390132)100:0.245016,(Deltaproteobacteria bacterium
RIFCSPHIGHO2_02_FULL_40_28__Deltaproteobacteria_Proteobacteria_Bacteria:0.1324667156,Deltaproteobacteria bacterium
GWA2_45_12__Deltaproteobacteria_Proteobacteria_Bacteria:0.1281335154)100:0.058937)71:0.040673,Deltaproteobacteria bacterium
GWA2_50_8__Deltaproteobacteria_Proteobacteria_Bacteria:0.2599246407)60:0.022501,((((Deltaproteobacteria bacterium
CG11_big_fil_rev_8_21_14_0_20_49_13__Deltaproteobacteria_Proteobacteria_Bacteria:0.1471892094,((Deltaproteobacteria bacterium
CG11_big_fil_rev_8_21_14_0_20_47_16__Deltaproteobacteria_Proteobacteria_Bacteria:0.1542711604,Deltaproteobacteria bacterium
RIFCSPLOWO2_02_FULL_46_8__Deltaproteobacteria_Proteobacteria_Bacteria:0.1915122991)35:0.013831,(Deltaproteobacteria bacterium
RIFOXYA12_FULL_58_15__Deltaproteobacteria_Proteobacteria_Bacteria:0.2295464401,(Deltaproteobacteria bacterium HGW-Deltaproteobacteria-
22__Deltaproteobacteria_Proteobacteria_Bacteria:0.2269099291,Sorangium
cellulosum__Sorangium_Polyangiaceae_Sorangiineae_Myxococcales_Deltaproteobacteria_Proteobacteria_Bacteria:0.1934235167)100:0.073812)34:0.038088)31:0.023290)39:0.036256,
Deltaproteobacteria bacterium GWA2_38_16__Deltaproteobacteria_Proteobacteria_Bacteria:0.2505789521)80:0.011482,Deltaproteobacteria bacterium
CG2_30_63_29__Deltaproteobacteria_Proteobacteria_Bacteria:0.2922298236)92:0.042038,Deltaproteobacteria bacterium
RIFOXYA12_FULL_61_11__Deltaproteobacteria_Proteobacteria_Bacteria:0.2629192672)56:0.014781)89:0.034538,Deltaproteobacteria bacterium
RBG_16_64_85__Deltaproteobacteria_Proteobacteria_Bacteria:0.3622680686)84:0.039905)77:0.025891,(((((((Deltaproteobacteria bacterium
GWA2_54_12__Deltaproteobacteria_Proteobacteria_Bacteria:0.1581972302,Deltaproteobacteria bacterium
RBG_19FT_COMBO_52_11__Deltaproteobacteria_Proteobacteria_Bacteria:0.1946167818)93:0.062909,(Deltaproteobacteria bacterium
CG_4_10_14_0_8_um_filter_43_12__Deltaproteobacteria_Proteobacteria_Bacteria:0.1701002973,(Syntrophaceae bacterium
CG2_30_49_12__Syntrophaceae_Syntrophobacterales_Deltaproteobacteria_Proteobacteria_Bacteria:0.0665133599,Syntrophus
gentianae__Syntrophus_Syntrophaceae_Syntrophobacterales_Deltaproteobacteria_Proteobacteria_Bacteria:0.0922717722)100:0.247682)96:0.036871)85:0.021404,(Desulfomonile
tiedjei__Desulfomonile_Syntrophaceae_Syntrophobacterales_Deltaproteobacteria_Proteobacteria_Bacteria:0.2197415918,Nitrospirae bacterium
CG2_30_53_67__Nitrospirae_Bacteria:0.1920216297)99:0.066751)83:0.013422,Candidatus Adiutrix intracellularis__Candidatus
Adiutrix_Deltaproteobacteria_Proteobacteria_Bacteria:0.2402750606)81:0.011450,(((Treponema
denticola__Treponema_Spirochaetaceae_Spirochaetales_Spirochaetes_Bacteria:0.1891072552,Salinispira
pacifica__Salinispira_Spirochaetaceae_Spirochaetales_Spirochaetes_Bacteria:0.2330715326)46:0.053545,((Treponema
medium__Treponema_Spirochaetaceae_Spirochaetales_Spirochaetes_Bacteria:0.0043182815,Treponema sp. OMZ
838__Treponema_Spirochaetaceae_Spirochaetales_Spirochaetes_Bacteria:0.0000026402)84:0.013638,Treponema
vincentii__Treponema_Spirochaetaceae_Spirochaetales_Spirochaetes_Bacteria:0.0038850260)99:0.109471)100:0.424313,(((((((candidate division TA06 bacterium 32_111__candidate
division TA06_Bacteria:0.3279608007,bacterium Candidatus Stahlbacteria CG08_land_8_20_14_0_20_40_26 Bacteria.__:0.4778332100)82:0.109378,candidate division TA06 bacterium
DG_24__candidate division TA06_Bacteria:0.1834356653)73:0.032908,candidate division Zixibacteria bacterium SM23_81 Bacteria.__:0.1713383481)74:0.050475,((Chlorobi bacterium
OLB4__Chlorobi_Bacteria:0.3993155556,Candidatus Edwardsbacteria bacterium RifOxyC12_full_54_24__Candidatus Edwardsbacteria_Bacteria:0.3513310629)44:0.027065,(Candidatus
Marinimicrobia bacterium CG_4_10_14_0_2_um_filter_48_9__Candidatus Marinimicrobia_Bacteria:0.4229485787,(Candidatus Desantisbacteria bacterium CG1_02_38_46__Candidatus
Desantisbacteria_Bacteria:0.0720381704,Candidatus Desantisbacteria bacterium CG1_02_49_89__Candidatus
Desantisbacteria_Bacteria:0.1122155838)100:0.262259)59:0.043578)69:0.030600)86:0.035820,Candidatus Handelsmanbacteria bacterium RIFCSPLOWO2_12_FULL_64_10__Candidatus
Handelsmanbacteria_Bacteria:0.4002748422)54:0.043873,candidate division KSB1 4572_119__candidate division KSB1_Bacteria:0.2494705240)53:0.031613,(Omnitrophica WOR_2
bacterium RIFCSPHIGHO2_02_FULL_67_20__Candidatus Omnitrophica_Bacteria:0.4009188528,candidate division BRC1 bacterium HGW-BRC1-1__candidate division
BRC1_Bacteria:0.5080218811)72:0.091161)47:0.052189)82:0.061434)72:0.015326,(Deltaproteobacteria bacterium
GWA2_47_9__Deltaproteobacteria_Proteobacteria_Bacteria:0.2214337505,(((Deltaproteobacteria bacterium
RIFOXYD12_FULL_57_12__Deltaproteobacteria_Proteobacteria_Bacteria:0.1306269046,((Desulfobulbus propionicus DSM
2032__Desulfobulbus_Desulfobulbaceae_Desulfobacterales_Deltaproteobacteria_Proteobacteria_Bacteria:0.1651982890,Desulfobulbaceae bacterium
A2__Desulfobulbaceae_Desulfobacterales_Deltaproteobacteria_Proteobacteria_Bacteria:0.0887179335)100:0.059141,uncultured Desulfofustis sp. PB-SRB1__environmental
samples_Desulfofustis_Desulfobulbaceae_Desulfobacterales_Deltaproteobacteria_Proteobacteria_Bacteria:0.2742562389)99:0.043148)100:0.132386,Desulfurivibrio
alkaliphilus__Desulfurivibrio_Desulfobulbaceae_Desulfobacterales_Deltaproteobacteria_Proteobacteria_Bacteria:0.1737932720)100:0.139995,Silvanigrella
aquatica__Silvanigrella_Silvanigrellaceae_Silvanigrellales_Oligoflexia_Proteobacteria_Bacteria:0.4280545308)63:0.029849)75:0.045679)73:0.015898,((Desulfobacca
acetoxidans__Desulfobacca_Syntrophaceae_Syntrophobacterales_Deltaproteobacteria_Proteobacteria_Bacteria:0.0872884193,Deltaproteobacteria bacterium
CG07_land_8_20_14_0_80_60_11__Deltaproteobacteria_Proteobacteria_Bacteria:0.1414834968)100:0.049874,(((((Syntrophobacter
fumaroxidans__Syntrophobacter_Syntrophobacteraceae_Syntrophobacterales_Deltaproteobacteria_Proteobacteria_Bacteria:0.0879689584,Syntrophobacteraceae bacterium
CG2_30_61_12__Syntrophobacteraceae_Syntrophobacterales_Deltaproteobacteria_Proteobacteria_Bacteria:0.1672422678)99:0.021217,Thermodesulforhabdus
norvegica__Thermodesulforhabdus_Syntrophobacteraceae_Syntrophobacterales_Deltaproteobacteria_Proteobacteria_Bacteria:0.1005898737)100:0.087155,Desulfobacteraceae
bacterium__Desulfobacteraceae_Desulfobacterales_Deltaproteobacteria_Proteobacteria_Bacteria:0.1658513963)83:0.039633,delta proteobacterium
NaphS2__Deltaproteobacteria_Proteobacteria_Bacteria:0.2175088750)79:0.019620,((Desulfobacterales bacterium
C00003060__Desulfobacterales_Deltaproteobacteria_Proteobacteria_Bacteria:0.1620010079,(Desulfatibacillum alkenivorans AK-
01__Desulfatibacillum_Desulfobacteraceae_Desulfobacterales_Deltaproteobacteria_Proteobacteria_Bacteria:0.1053438653,(Deltaproteobacteria bacterium
RBG_13_49_15__Deltaproteobacteria_Proteobacteria_Bacteria:0.1169203918,Deltaproteobacteria bacterium
SG8_13__Deltaproteobacteria_Proteobacteria_Bacteria:0.1971839586)100:0.083459)99:0.037957)99:0.054001,Desulfobacterales bacterium
C00003106__Desulfobacterales_Deltaproteobacteria_Proteobacteria_Bacteria:0.1713345599)99:0.040460)97:0.029192)71:0.019362)65:0.009598)59:0.005929,(Desulfarculus
baarsii__Desulfarculus_Desulfarculaceae_Desulfarculales_Deltaproteobacteria_Proteobacteria_Bacteria:0.0427871644,Desulfocarbo
indianensis__Desulfocarbo_Desulfarculaceae_Desulfarculales_Deltaproteobacteria_Proteobacteria_Bacteria:0.1430210482)100:0.127853)40:0.005963,((((candidate division KSB3
bacterium__candidate division KSB3_Bacteria:0.2718774751,((((Acidobacteriaceae bacterium KBS
96__Acidobacteriaceae_Acidobacteriales_Acidobacteria_Bacteria:0.0541000245,Bryobacter
aggregatus__Bryobacter_Bryobacteraceae_Solibacterales_Solibacteres_Acidobacteria_Bacteria:0.0911619013)100:0.020931,Candidatus Solibacter usitatus__Candidatus
Solibacter_Solibacteraceae_Solibacterales_Solibacteres_Acidobacteria_Bacteria:0.0488250844)100:0.066670,Chloracidobacterium
thermophilum__Chloracidobacterium_Blastocatellia_Acidobacteria_Bacteria:0.2631521082)89:0.032984,(Acidobacteria bacterium
13_1_40CM_56_16__Acidobacteria_Bacteria:0.1079176011,((((Acidobacteria bacterium 13_1_40CM_2_64_6__Acidobacteria_Bacteria:0.0312824685,((Acidobacteria bacterium
RIFCSPLOWO2_02_FULL_67_36__Acidobacteria_Bacteria:0.0846616797,(Acidobacteria bacterium 13_1_40CM_4_65_8__Acidobacteria_Bacteria:0.0400292146,Acidobacteria bacterium
RIFCSPLOWO2_02_FULL_64_15__Acidobacteria_Bacteria:0.0342133810)100:0.017232)100:0.013185,Acidobacterium
sp.__Acidobacterium_Acidobacteriaceae_Acidobacteriales_Acidobacteria_Bacteria:0.1187126300)100:0.011827)100:0.048699,Acidobacteria bacterium SCN 69-
37__Acidobacteria_Bacteria:0.0607420381)100:0.041316,Luteitalea pratensis__Luteitalea_Acidobacteria subdivision 6_Acidobacteria_Bacteria:0.1537071063)98:0.026824,Acidobacteria
bacterium Mor1__Acidobacteria subdivision 22_Acidobacteria_Bacteria:0.4377377382)82:0.031578)64:0.017705)70:0.046524)77:0.024645,(Acidobacteria
bacterium__Acidobacteria_Bacteria:0.3482349585,Geothrix
fermentans__Geothrix_Holophagaceae_Holophagales_Holophagae_Acidobacteria_Bacteria:0.4421640344)85:0.142677)89:0.028170,(Nitrospinae bacterium
CG11_big_fil_rev_8_21_14_0_20_56_8__Nitrospinae_Nitrospinae/Tectomicrobia group_Bacteria:0.2050183352,Candidatus Tectomicrobia bacterium
RIFCSPLOWO2_02_FULL_70_19__Candidatus Tectomicrobia_Nitrospinae/Tectomicrobia group_Bacteria:0.2938620159)100:0.091709)90:0.056499,((Nitrospirae bacterium
RIFCSPHIGHO2_01_FULL_66_17__Nitrospirae_Bacteria:0.1280805528,Nitrospirae bacterium
RIFCSPLOWO2_12_FULL_63_8__Nitrospirae_Bacteria:0.1913097410)99:0.097974,Leptospirillum
ferriphilum__Leptospirillum_Nitrospiraceae_Nitrospirales_Nitrospirae_Bacteria:0.3476712558)100:0.103313)40:0.022361)38:0.017477,bacterium BMS3Abin14
Bacteria.__:0.2963687996)77:0.028820,(Deltaproteobacteria bacterium RBG_13_65_10__Deltaproteobacteria_Proteobacteria_Bacteria:0.1732689782,Syntrophorhabdus
aromaticivorans__Syntrophorhabdus_Syntrophorhabdaceae_Syntrophobacterales_Deltaproteobacteria_Proteobacteria_Bacteria:0.3248785955)89:0.029314)83:0.060062)98:0.058281)
99:0.304507)100:0.124960,Eutreptiella gymnastica NIES-381:0.2337393534)100:0.459214);
